# [Retracted] Genotoxicity of chloroacetamide herbicides and their metabolites *in vitro* and *in vivo*

**DOI:** 10.3892/ijmm.2025.5511

**Published:** 2025-03-04

**Authors:** Xinyan Ma, Ying Zhang, Mingyang Guan, Weidong Zhang, Huifang Tian, Caixiao Jiang, Xiaoxin Tan, Weijun Kang

Int J Mol Med 47: 103, 2021; DOI: 10.3892/ijmm.2021.4936

Following the publication of the above paper, it was drawn to the Editor's attention by a concerned reader that certain of the JNK and phosphorylated (pho)-JNK western blotting data shown in Fig. 8 on p. 7 were strikingly similar to data featured in a pair of other articles written by different authors at different research institutes, one of which (submitted to the journal *Molecular Medicine Reports*) has been subsequently retracted, whereas the other (submitted to the journal *Renal Failure*) was received at that journal prior to the receipt of the above article at *International Journal of Molecular Medicine*.

In view of the fact that the abovementioned data had already apparently been submitted elsewhere prior to the receipt of this paper at *International Journal of Molecular Medicine*, the Editor has decided that this paper should be retracted from the Journal. The authors were asked for an explanation to account for these concerns, but the Editorial Office did not receive a reply. The Editor apologizes to the readership for any inconvenience caused.

